# Pinnipeds and PTSD: An Analysis of a Human-Animal Interaction Case Study Program for a Veteran

**DOI:** 10.1155/2018/2686728

**Published:** 2018-06-13

**Authors:** Rachel A. Wortman, Theresa Vallone, Michele Karnes, Christine Walawander, Dion Daly, Bonnie Fox-Garrity

**Affiliations:** Department of Occupational Therapy, D'Youville College, 320 Porter Avenue, Buffalo, NY 14201, USA

## Abstract

The objective of this study was to examine the impact of a pinniped (grey and harbor seals) facilitated human-animal interaction pilot program on the self-reported PTSD-like symptoms of a veteran. This study analyzed preexisting, deidentified data that represented the participant's scores on the Post-Traumatic Stress Disorder Checklist (PCL-5). The PCL-5 was completed as part of a pilot program operated in partnership between the Veteran and Military Affiliated Research Center (VMARC) and a local aquarium. Scores on the PCL-5 were collected prior to (T1), midway (T2), and immediately after (T3) completion of the *Project Seal to Heal* program. Changes in the scores of each item were reported for the participant, for aggregated items that represented different clusters of PTSD symptoms, and for overall scores. Results revealed decreased scores in 11 of the 20 PTSD symptom-related items, improvement in the sum scores for each criteria symptom cluster, and a 15-point decrease in the overall PCL-5 score, indicating clinical significance. These results serve as a call to motivate future research investigating pinniped interactions with veterans who have PTSD in order to determine therapeutic clinical application and outcomes.

## 1. Introduction

In 2013, the World Health Organization (WHO) published their Mental Health Action Plan for 2013–2020. In this report, WHO claims that there is “no health without mental health” (p. 6). Globally, it is estimated that one-fourth of families will be directly affected with at least one member of that family identifying as having a mental or behavior disorder. These effects are not only experienced by the individual and his or her loved ones, but by society overall [1, 2]. The United States' National Institute of Mental Health (NIMH) has projected that by 2030; global spending directly related to mental health will equal $6.0 trillion, in comparison to the 2010 global spending cost of $2.5 trillion. Moreover, poor mental health may perpetuate, or be perpetuated by, maladaptive behavior [3, 4]. These mutually influential healthcare concerns often occur in conjunction with other disorders and/or impairments, making them two of the largest contributors of global economic burden [3, 5].

One such mental health ailment that often alters behavior is posttraumatic stress disorder, or PTSD, which is currently defined as a stress disorder brought on by exposure to a traumatic event. As such, PTSD falls into the mental and behavioral health spectrum [6]. According to the WHO, approximately 3.6% of the world population is afflicted with PTSD. An estimated 7.0–8.0% of the US population contribute to that percentage [6–10]. Though there are many populations exposed to traumatic situations, the prevalence of PTSD within the United States military veteran population has not only increased public awareness of the disorder but has also reached astonishing levels.

The US Department of Veterans Affairs (USDVA) and the National Center for PTSD estimates that 11–20% of military personnel involved in Operations Iraqi Freedom (OIF) and Enduring Freedom (OEF) have been diagnosed with PTSD. This estimate does not include veterans who were involved in WWII, the Vietnam War, or the Gulf War [8]. Due to the overtaxing of specific mental capacities, veterans with PTSD have also been recognized to be more likely than those with other mental health diagnoses to demonstrate high-risk drug use and adverse clinical outcomes, such as injuries and overdoses [11]. Comorbidities such as depression, anxiety, and difficulty sleeping often accompany PTSD and can affect nearly every activity and interaction with which individuals engage. Despite growing trends of PTSD diagnosis within military populations, effective treatments to combat the disorder within this sector of the population have not followed suit [12].

Currently, the majority of veterans diagnosed with PTSD are treated with serotonin reuptake inhibitor antidepressants (SSRIs). However, SSRIs have been found to contribute little more than a placebo in the alleviation of mild to moderate symptoms [12, 13]. In fact, in 2007 it was reported that sertraline, an SSRI, provided no additional benefits when compared to a placebo, specifically when used by veterans who had PTSD [14]. Yet, seven years later in 2012, 89% of the US veterans who had a diagnosis of PTSD were treated with SSRIs. Simultaneously less than 20% of veterans who were diagnosed with PTSD also reported receiving adequate treatment, no matter the treatment type [15]. A diagnosis of PTSD often comes with the recommendation for psychotherapy, but in 2016, it was reported that only one-third of the troops who had identified as having PTSD went on to receive the minimum number of therapeutic sessions suggested after receiving their diagnosis. This was either due to personal choice or because there was a current shortage of healthcare professionals available to address the growing demand and backlog for the treatment of PTSD for veterans [16, 17]. The implication is that many veterans with PTSD either are not receiving treatment or are paying for a treatment that is ineffective.

This information notwithstanding, Global War on Terror (GWOT) veterans are making more claims for injury and/or illness than any veteran population of previous wars, and peak cost spent on veteran medical care specific to disability does not occur until decades after a military member has been discharged. For post-9/11 veterans discharged in 2013, it has been estimated that costs linked to medical treatment will exceed $1.0 trillion by 2053. Due to these unprecedented increases in veterans seeking assistance, it has been postulated that as of 2016, veteran healthcare cost approximations established by the US Department of Defense (DOD) are low [16]. Without exploration into new avenues of PTSD treatment alternatives, a perpetuation of massive economic, social, and environmental burdens will continue to cultivate [12, 16].

Global appreciation of the gravity and impact of mental health on economics and human well-being continues to expand. Analogously, a rise in new approaches to address these ailments has been steadily investigated. As human well-being is multifaceted, with many instrumental and contingent components, contrived inquiry into the establishment and maintenance of this well-being is beset with a multitude of complex challenges [18]. The investigation into methods of creatively addressing burdens linked to PTSD presents a unique opportunity for healthcare professionals.

Currently, there is an intercontinental proliferation of organizations compelling healthcare professional consciousness in the direction of new and innovative approaches for human health and well-being, simultaneously uniting global human constitution and obligations to conserve a salubrious planet with advantageous economic applications. Ecology, biodiversity, and environmental sustainability have all been recognized as essential underpinnings to public and environmental health. Changes in environments, including changes in organisms comprising those environments, can potentially directly and indirectly impact human health [19]. Few healthcare professions have the capacity to fully incorporate the necessity of a true holistic approach to human well-being, accounting for the complex and dynamic relationships between environments, behaviors, and overall health. However, occupational therapy stands as an aberration against this inclination.

In truth, occupational therapists stand at the precipice of utilizing their unique expertise to not only aid individuals but also, in turn, affect positive influences in global economics, ecological sustainability, and community well-being. This conviction has been expressed in the World Federation of Occupational Therapy's (WFOT) position statement on environmental sustainability. The WFOT takes the position that occupational therapists should not only attend to individual needs but also simultaneously promote well-being and environmental health [20]. Occupational therapists have the expertise and responsibility to recognize humans as both influencers of their environment and beings whom are influenced by their environment [18, 21].

It is vital that occupational therapists are cognizant of ecology and biodiversity, or the diversity of life, while fulfilling their core role addressing occupations and occupational performance. This is due to the fact that human occupations are transformed in response to the availability and condition of environmental resources. Efforts within the profession should be directed toward environmental sustainability while paralleling collaborative efforts directed toward optimal occupational performance of clients and communities. These efforts should also meet growing healthcare concerns within specific populations. Occupational therapists specialize in engaging a single component from an environment as the tool to facilitate a change in unhealthy or maladaptive human behavior. Biodiversity may serve as both the tool and the objective for augmented health. However, addressing all of the issues that accompany global shifts in ecology and biodiversity, while simultaneously addressing immediate healthcare demands, such as the increased burden of mental illnesses, can be daunting.

The Model of Human Occupations (MOHO) and the biophilia hypothesis serves as guiding conceptual frameworks for both mental health and as a tool for environmentally conscious healthcare practices, endorsing the dynamic interplay between organisms and their environment. MOHO is an occupational therapy-specific framework that encompasses the idea that occupational engagement is an open and dynamic system of interaction and change. Not only is the person seen as an input mechanism, and the occupation as the output result, but MOHO considers the environmental influence on the motivation, patterns, and performance of the individual [22]. Much as MOHO claims that humans have a need to engage in activities within their environments, biophilia attests that nature contains components that support life and that humans have a want and need to interact with natural organisms, as they are a part of the “self,” meaning a part of the person [23–28]. Under the scope of these theories, emerging therapeutic practices have sprouted, including animal-assisted therapy (AAT) [27, 29].

Health benefits associated with AAT have started to be explored and have proven promising, with the bulk of AAT research focusing on domesticated, companion animals [30]. Inquiries into the efficacy of nonhuman animals in therapeutic interventions beyond the scope of companionship have begun to emerge, but are relatively limited. Central to these investigations is the realization that all humans are continuously influenced by, and dependent upon, nonhuman animals. Purportedly, nonhuman animals influence every day occupations, as they are, according to Huggett [31], a part of the “entirety of life” and, as such, are also a part of life support systems. This is an inherent virtue of AAT, the application of a component of life in a therapeutic capacity, in order to better that life, engaging biodiversity as the means and the end.

However, increased research of human-animal interactions (HAIs) is obligatory for therapeutic understanding of the potential success of AAT. There is also a need for new medicinal techniques, utilizing information garnered from the HAI research that concomitantly benefits the human client, while acknowledging the importance of an explicit nonhuman participant. To truly recruit a component of “life” as a therapeutic instrument in AAT, nonhuman animals that influence environmental health are required. Conceivably, many of these animals are wild and considered vulnerable in their relationship with humans, or made so indiscreetly through the impact of human behavior on their environment. Investigating HAIs, beyond companion animals, and with vulnerable nonhuman populations may prove to be beneficial [31].

According to the Millennium Ecosystem Assessment (MEA) [32], vulnerability is defined as “exposure to contingencies and stress, and the difficulty in coping with them” (p. 605). There are research articles and opinion publications addressing the potential employment of certain nonhuman animals in AAT which may fall under the scope of vulnerable, specifically aquatic animals [31, 33]. Aquatic animals are of special consideration for AAT application, as their health and well-being is often influenced by human activity and, in turn, ultimately an influencer on global human health [34].

The employment of oceanic mammals in therapeutic interactions has potential, as these nonhuman animals are under great ecological shifts amidst limited public awareness about such. In general, aquatic animals, when discussed in AAT research, are almost exclusively limited to bottlenose dolphins, a practice referred to as dolphin-assisted therapy (DAT) [35, 36]. Though DAT may be tangential to AAT in an aquatic environment, the engagement of pinnipeds (seals, sea lions, fur seals, and walrus) in a similar capacity has until now gone unexamined.

Pinnipeds play an important role in global biodiversity and environmental health [37–39]. When compared to dolphins, seals and sea lions are much more commonly rescued and kept in captivity when injured. These rescues, as well as human raise pinnipeds, may be considered a vulnerable population in need of care and are abundantly more accessible to the general public than dolphins [40–42]. Demeanor, accessibility, and other distinct characteristics among pinnipeds make them apt candidates for employment in AAT. Pinniped engagement in AAT, a possible alternative to DAT, has yet to be investigated, in any capacity, for therapeutic benefit in human populations [43–45].

Currently, there is a lack of research addressing the impact of interactions between veterans with PTSD-like symptoms and rescued or otherwise nonrelease seals. By utilizing the MOHO and the biophilia hypothesis in combination, this research sought to evaluate a veteran-pinniped HAI program at a local aquarium in order to identify (1) the impact of the pinniped HAI program for veterans identifying as having PTSD-like symptoms and (2) the implications for future occupational therapy research specific to pinniped HAIs.

## 2. Methods

Upon approval by the D'Youville College's Internal Review Board (IRB), the researcher requested use of a deidentified data set from the Veteran and Military Affiliated Research Center (VMARC) at a small private, liberal arts college in Buffalo, NY. The deidentified data was drawn from preexisting information gathered by the VMARC pertaining to a pinniped facilitated HAI program entitled *Project Seal to Heal*. This program was conducted by the VMARC in partnership with the Aquarium of Niagara Falls and included aquarium staff, a representative from the VMARC with knowledge and experience in PTSD mediation, and four seals, including one rescued grey seal, one rescued Pacific harbor seal, one rescued Atlantic harbor seal, and one harbor seal who was raised under human care.

*Project Seal to Heal* participants were introduced to only one of the four seals per session, with each session incorporating a different seal. Each session was in total one hour in duration, which encompassed the total time spent at the Aquarium. Participants were allotted direct and indirect interactions with the seals. Indirect interactions were educationally based, ranging in scope from broad to narrow topics. Broad topics included education regarding ocean ecology, pinniped taxonomy, pinniped distribution and habitat, pinniped anatomy and physiology, and conservation themes such as human/pinniped interface and implications. Narrow topics included education in husbandry tasks, which involves the actions required in order to care for wild animals in captivity, such as preparing meals for the seals and seal enrichment activities. Direct interactions were provided when participants shared space with and/or were in physical contact with the seals. Each participant was afforded a minimum of ten minutes of direct interaction with the seal, per session during which information was provided regarding the seal's personal history, preferences, and dislikes. Participants were involved in training the seal of that session, which included feeding, touching, and observing the seal's behavior.

Additionally, participants completed a self-reported questionnaire regarding their subjective rating of PTSD symptoms. These questionnaires were collected by and stored with the VMARC for program evaluation. No demographic or personal information beyond the PCL-5 scores were recorded on the deidentified data form, in order to maintain anonymity for the researcher. The specific questionnaire utilized during the program was the PTSD Checklist (PCL-5) (the appendix).

### 2.1. PCL-5

The PTSD checklist (PCL) is currently one of the most widely utilized self-report measurement tools for PTSD. The most contemporary version of the PCL-5 is used as a resource for a variety of purposes in unison with the fifth edition of the Diagnostic and Statistical Manual of Mental Disorders (DSM-5), including monitoring changes in symptoms related to PTSD during a range of treatment techniques and research [8, 46, 47]. As a self-report instrument, the PCL-5 contains 20 items measured on a Likert scale ranging from zero to four. The lowest score any one individual can receive is a zero, which would indicate no PTSD symptoms at all. The highest overall score that an individual can receive is an eighty, denoting severe and impactful symptoms of PTSD [46, 48]. The scoring to the items is organized parallel to the cluster criteria of PTSD symptoms. Questions one to five are related to PTSD diagnostic criterion B, questions six and seven are linked to criterion C, questions eight to fourteen align with criterion D, and questions fifteen to twenty are associated with criterion E. The PCL-5 has been established as a sound instrument for the measurement of PTSD symptoms, with an internal consistency of *α* = 0.94, a test-retest reliability of *r* = 0.82, a convergent validity of *r* = 0.74 to 0.85, and a discriminant validity of *r* = 0.31 to 0.60 [46, 49]. When used for estimating changes in symptoms due to an intervention, a reduction of five points or more reflects a reliable statistical change, where a reduction of ten points or more is an indication of clinical significance [48, 50, 51].

### 2.2. Treatment of Data

Participants in *Project Seal to Heal* completed the PCL-5 at T1, T2, and T3 equating to three sets of scores. These were supplied to the researcher via an encrypted, electronic spreadsheet by the VMARC (Table 1). Prior to the transfer of this information, the VMARC changed the names of the program participants to a number code in order to ensure anonymity for this research. Demographic information was also not provided on the spreadsheet.

A range of numerical responses one to four corresponded to the Likert scale for the PCL-5. Zero corresponded to an answer of “not at all,” one corresponded to an answer of “a little bit,” two corresponded to “moderate,” three correlated to “quite a bit,” while an answer of four corresponded to “extremely.” The raw data provided consisted of participant responses to the 20 questions on the PCL-5 for each time period in which data was collected. The data existed in a column that was designated by a “T” identifier corresponding to each data collection point, meaning “T1” represented the pretest data collected in week one, “T2” represented the midpoint data, and “T3” represented the posttest data. Pertinent data gained from the study was reported utilizing tables and graphs for easy comprehension of alterations in reported PTSD-like symptoms before, during, and after interactions with rescued seals.

From this information, the researcher calculated the difference in reported scores for each week the questionnaires were administered (Table 1). To do this, the researcher subtracted T2 from T1 in order to obtain the difference in reported scores from pretest to midpoint test. The researcher also subtracted T3 from T2 and from T1 in order to obtain the difference in reported scores for midpoint test to posttest, as well as from pretest to posttest. Any calculated differences greater than zero indicated that the participant had responded positively to the HAI for the specific question and the symptom corresponding to that question. Calculated differences less than zero indicated a negative response to the HAI, and a score of zero indicated no noticeable change to in the reported symptom. The calculated differences in scores could range from a 4.0 to a −4.0 (Table 1).

The calculated differences between T1, T2, and T3 were transferred to Table 2 by the researcher in order to tally an aggregate mean for each item on the PCL-5. From these means, the researcher determined if the symptom correlating to each of these PCL-5 items was influenced positively (a decrease in the aggregated symptom score), negatively (an increase in the aggregated symptom score), or not at all (a reported aggregated mean of zero in the symptom score).

Table 3 shows the sum of scores that were calculated corresponding to the PCL-5 questions, that, when clustered, corresponded to PTSD diagnostic criteria for symptomatology. These questions were clustered as such: questions one to five were added for the sum related to criteria B (intrusion symptoms), questions six and seven were added for the sum related to criteria C (symptoms correlating to persistent reexperiencing of the stressful event), questions eight to fourteen were added for the sum related to criteria D (symptoms correlating to negative alterations in mood and cognition), and questions fifteen to twenty were added for the sum related to criteria E (symptoms correlating to alterations in arousal and reactivity).

The accumulative items for each cluster of questions were calculated for T1, T2, and T3. The overall difference between T1 and T3 were calculated, in order to determine changes in the PCL-5 question clusters for PTSD criteria scores reported prior to participation in the program when compared to scores reported after completion of the program.

Additionally, the overall PCL-5 scores were examined for each week the participant completed the PCL-5. The highest overall score any one participant could receive was an 80, indicating the highest severity of symptoms, and the lowest a score a zero, which would indicate no reported symptoms at all.

## 3. Results

At the onset of *Project Seal to Heal*, four veterans were screened by the VMARC for participation in the six-week program. The VMARC supplied the researcher with the results from one veteran (identified as participant number three) who completed the four weeks of *Project Seal to Heal.* Due to adverse weather conditions, the program itself was limited to four weeks, and three of the participants were unable to attend all four sessions.

### 3.1. Individual PLC-5 Item Scores

Table 1 summarizes the scores supplied by the VMARC for each week that the PCL-5 was administered, as well as the difference between each item of the PCL-5 from T1, T2, and T3. It was noted that the program participant reported gave the highest score of 4.0 at T1 in six of the twenty questions, indicating that he/she was “extremely” impacted by the PTSD symptoms correlating to that specific PCL-5 question. The participant also at T1 gave the second highest score of a three for seven of the PCL-5 questions, indicating that the participant was impacted “quite a bit” from the symptom correlating to the question.

A “moderate” rating for symptoms was given in four of the questions. The participant did not give a zero for any question, but did answer with a one for the remaining two questions, indicating the symptom impacted his/her life “a little bit.”

From these scores, the difference between each PCL-5 item score was calculated. These calculated scores were listed in the appropriate columns, labeled T1–T2, T2–T3, and T1–T3 as seen in Table 2, which illustrates the aggregated mean differences per item on the PCL-5. The mean illustrates the greatest areas of change in symptoms with consideration of the differences between data points weeks. Overall, there were two PCL-5 items found to slightly ($\bar{x}=0.667$) negatively impact the PTSD symptoms, meaning an increase in the reported score. These occurred in repeated, disturbing, and unwanted memories of the event and loss of interest in enjoyable activities. These symptoms were originally rated as having a little to a moderate impact on symptoms, respectfully. Seven PCL-5 items indicated no mean change in the symptom ($\bar{x}=0$). These included self-blaming for the stressful event, feeling distant or cut off from others, having difficulty experiencing feelings, irritable behavior, feeling irritable or easily startled, having difficulty concentrating, and having trouble falling asleep.

Nine of the PCL-5 mean item scores decreased, indicating an improvement in the symptom. This decreases ranged from $\bar{x}=0.667$ to $\bar{x}=2$, with the largest differential change ($\bar{x}=2$) reported in symptoms related to suddenly feeling or acting as if the stressful event were occurring again, feeling very upset after experiencing a reminder of the stressful event, avoiding memories, thought, or feelings related to the stressful experience.

### 3.2. Criteria Symptom Clusters and Overall PCL-5

Table 3 illustrates sums of and alterations in the reported scores correlating to each cluster of symptom composing the PTSD criteria recorded for T1, T2, and T3, as well as the overall difference from T1 to T3. From T1 to T3, results indicate that all criteria clusters reduced in the reported score, with the most significant reductions in score occurring in criteria B, intrusion (reduction of six points), and criteria C, persistent avoidance (reduction of five points). A reduction of two points was reported for criteria D (negative alterations in cognition and mood) and criteria E (alterations in arousal and reactivity). Though the total criteria scores all reduced, a deviation occurred in the reported symptoms of criteria D, in which the T3 score increased in comparison to the T2 score. However, criteria D at T3 remained lower than the T1 score. Further, Tables 1 and 2 show the overall score of the PCL-5 reduced from T1 to T2 by eleven points and from T2 to T3 by four points. This accounts for a reduction of fifteen points for the overall PCL-5 score.

## 4. Discussion

Previous research focusing on the engagement of nature or natural elements in order to address a variety of ailments have shown physiological, psychosocial, emotional, and mental benefits for the individuals who participated in those studies [12, 26, 52–57]. Specific to HAI, previous studies have found benefits in decreased symptoms of depression and anxiety, increased self-esteem, positive alterations in behavior, decreased feelings of isolation, increased feelings of empathy, increase in positive outlook, decrease in irritability, and an overall increase in perceived quality of life [6, 30, 58–63]. The results of this study support these previous findings with regard to the potential merit of HAIs for the veterans who have PTSD.

Results indicate that overall, participation in *Project Seal to Heal* was clinically significant in the reduction of PTSD-like symptoms for the veteran participant who completed the program. Comparisons of PCL-5 items for each cluster indicated the greatest positive impact from program participation for criteria B and C (intrusion and persistence). The specific symptoms most significantly and positively impacted included those of suddenly feeling or acting as if the stressful experience were occurring again; feeling very upset when reminded of the stressful event; avoiding memories, thoughts, or feelings related to the stressful experience; and avoiding external reminders of the stressful experience.

Though the majority of PCL-5 items indicated a decrease in the correlating symptoms, seven of the items indicated no change in the mean scores. This indicates that participation in the program possibly had no effect on those specific symptoms. These symptoms included blaming the self for the stressful event, feeling distant or cut off from others, having trouble experiencing positive feelings, experiencing irritable behavior and/or angry outbursts, feeling jumpy or easily startled, having difficulty concentrating, and having trouble falling or staying asleep.

The symptom related to PCL-5 item nineteen (difficulty concentrating) was of the most interest, as this item was reported as a four, or extremely disruptive in everyday life, for T1, T2, and T3. Additionally, item thirteen (feeling distant or cut off from other people) was initially reported as a four at T1, dipping to a two at T2, and then rising again to a four at T3. These items were the only two without an overall change of four when comparing T1 to T3. Although the items decreased only to return to the previously reported level, the implication is that participation in *Project Seal to Heal* may have no impact on that symptom when the symptom is initially reported at the highest level of severity.

There were also reported negative impacts on symptoms including those of repeated, disturbing, and unwanted memories of the stressful event and loss of interest in enjoyable activities (items one and twelve). However, the overall positive impact indicated on the PCL-5 suggests not only that *Project Seal to Heal* was clinically significant in the reduction of symptoms for the veteran participant but also that pinniped (seal) facilitated human-animal interaction programs merit further investigation by and within the therapeutic community.

## 5. Limitations

For this study, there were several limitations. The single participant sample size, lack of a control group, and no randomization make it impossible to generalize the results of the study. Another limitation is that only participants who are interested in seals were likely to partake in *Project Seal to Heal*. This may lead to potential bias in reported symptom alterations as individuals who have a positive outlook on animals may already have a predisposition to receive or experience a change in their ailment. Additionally, a limitation lies in the fact that the participant only had interactions with captive grey and harbor seals; thus, the results cannot be generalized to all pinnipeds. Beyond that, this study is limited by the cultural beliefs of the geological location, inland northern East Coast United States, and the language of that community when specifically addressing and interacting with marine environments and pinnipeds.

## 6. Conclusion

The results found in this study indicate that participation in the *Project Seal to Heal* program was clinically significant for the reduction of PTSD-like symptoms for the veteran who completed the program. The PTSD symptoms corresponding to the diagnostic criteria that were most notably impacted were those related to intrusion and persistence in reliving the stressful event. However, participation in the program had a negative noted effect on two symptoms and no noticeable impact on six symptoms. The results of the study cannot be generalized due to small sample size, no control group, limited pinniped species inclusion, and no randomization. The findings do support continued research in pinniped facilitated HAI in order to address the growing burden of PTSD within the veteran community. The findings are of interest to occupational therapists as engagement of this particular animal in a therapeutic intervention may be advantageous in addressing global healthcare needs in mental health, as well as attending to the WFOT's call for practitioners to address immediate healthcare demands while simultaneously confronting environmental health.

## Figures and Tables

**Table 1:** Program seal to heal PCL-5 scores for T1, T2, and T3 (*n* = 1).

Participant number	PCL question number	PCL (T1)	PCL (T2)	(T1–T2)	PCL (T3)	(T2–T3)	(T1–T3)
3	1	1	2	−1	2	0	−1
	2	2	3	−1	1	2	1
	3	4	1	3	1	0	3
	4	4	3	1	2	1	2
	5	3	2	1	2	0	1
	6	4	2	2	1	1	3
	7	3	1	2	1	0	2
	8	4	4	0	3	1	1
	9	3	2	1	2	0	1
	10	2	3	−1	2	1	0
	11	3	3	0	2	1	1
	12	2	1	1	3	−2	−1
	13	4	2	2	4	−2	0
	14	1	1	0	1	0	0
	15	3	2	1	3	−1	0
	16	3	2	1	2	0	1
	17	4	4	0	3	1	1
	18	2	2	0	2	0	0
	19	4	4	0	4	0	0
	20	3	4	−1	3	1	0
	**Sum**	**59**	**48**	**11**	**44**	**4**	**15**

Key to PCL answers: 0 = not at all, 1 = a little bit, 2 = moderately, 3 = quite a bit, 4 = extremely; key to test and week identification: T1 = week 1, T2 = week 2, T3 = week 4.

**Table 2:** PCL-5 aggregated mean ($\bar{x}$) item scores for the PCL-5 (*n* = 1).

PCL-5 question	T1–T2	T2–T3	T1–T3	Aggregate $\bar{x}$ [(T1–T2–T3)/3]	↑ $\bar{x}$ score	↓ $\bar{x}$ score	No change in $\bar{x}$ score
1	−1	0	−1	−0.667	X		
2	−1	2	1	0.667		X	
3	3	0	3	2		X	
4	1	1	2	2		X	
5	1	0	1	0.667		X	
6	2	1	3	2		X	
7	2	0	2	0.667		X	
8	0	1	1	0.667		X	
9	1	0	1	0.667		X	
10	1	1	0	0			X
11	0	1	1	0.667		X	
12	1	−2	−1	−0.667	X		
13	2	−2	0	0			X
14	0	0	0	0			X
15	1	−1	0	0			X
16	1	0	1	0.667		X	
17	0	1	1	0.667		X	
18	0	0	0	0			X
19	0	0	0	0			X
20	−1	1	0	0			X

Table key: T1 = week 1 (pretest), T2 = week 2 (midpoint), T3 = week 4 (posttest), $\bar{x}$ = mean, ↑ = increase, ↓ = decrease, X = indicated changed.

**Table 3:** Impact on PCL-5 accumulative sum (*Σ*) scores for PTSD criteria clusters and overall PCL-5 score (*n* = 1).

PTSD criteria	PCL-5 questions clusters for PTSD criteria	T1 Σ score	T2 Σ score	T3 Σ score	Overall difference from T1–T3 (week 1 to week 4)
B: Intrusion	1 to 5	14	11	8	−6
C: Persistent	6 and 7	7	3	2	−5
D: Negative alterations in cognition and mood	8 to 14	19	16	17	−2
E: Alterations in arousal and reactivity	15 to 20	19	18	17	−2
Total score	**1 to 20**	**59**	**48**	**44**	**−15**

Table key: T1 = week 1 (pretest), T2 = week 2 (midpoint), T3 = week 4 (posttest), Σ = sum (of cluster questions per PTSD criteria).

**Table 4:** 

In the past month, how much were, you bothered by	Not at all	A little bit	Moderately	Quite a bit	Extremely
(1) Repeated, disturbing, and unwanted memories of the stressful experience?	0	1	2	3	4
(2) Repeated, disturbing dreams of the stressful experience?	0	1	2	3	4
(3) Suddenly feeling or acting as if the stressful experience were actually happening again (as if you were actually back there reliving it)?	0	1	2	3	4
(4) Feeling very upset when something reminded you of the stressful experience?	0	1	2	3	4
(5) Having strong physical reactions when something reminded you of the stressful experience (e.g., heart pounding, trouble breathing, and sweating)?	0	1	2	3	4
(6) Avoiding memories, thoughts, or feelings related to stressful experience?	0	1	2	3	4
(7) Avoiding external reminders of the stressful experience (e.g., people, places, conversations, activities, objects, or situations)?	0	1	2	3	4
(8) Trouble remembering important parts of the stressful experience?	0	1	2	3	4
(9) Having strong negative beliefs about yourself, other people, or the world (e.g., having thoughts such as: I am bad, there is something seriously wrong with me, no one can be trusted, the world is completely dangerous)?	0	1	2	3	4
(10) Blaming yourself or someone else for the stressful experience or what happened after it?	0	1	2	3	4
(11) Having strong negative feelings such as fear, horror, anger, guilt, or shame?	0	1	2	3	4
(12) Loss of interest in activities that you used to enjoy?	0	1	2	3	4
(13) Feeling distant or cut off from other people?	0	1	2	3	4
(14) Trouble experiencing positive feelings (e.g., being unable to feel happiness or have loving feelings for people close to you)?	0	1	2	3	4
(15) Irritable behavior, angry outbursts, or acting aggressively?	0	1	2	3	4
(16) Taking too many risks or doing things that could cause you harm?	0	1	2	3	4
(17) Being “superalert” or watchful or on guard?	0	1	2	3	4
(18) Feeling jumpy or easily startled?	0	1	2	3	4
(19) Having difficulty concentrating?	0	1	2	3	4
(20) Trouble falling or staying asleep?	0	1	2	3	4

Source: US Department of Veteran Affairs (VA), National Center for PTSD, (2014). *PTSD checklist for DSM-5 (PCL-5)*. Retrieved from http://www.ptsd.va.gov/professional/assessment/adult-sr/ptsd-checklist.asp.

## Appendix

### The PTSD Checklist for DSM-5 (PCL-5) Self-Reported Questionnaire (Items One to Twenty)

*Instructions*: Table 4 shows a list of problems that people sometimes have in response to a very stressful experience. Please read each problem carefully and then circle the numbers to the right to indicate how much you have been bothered by that problem *in the past month*.
